# Supplementary material for: Mutations in PCYT2 disrupt etherlipid biosynthesis and cause a complex hereditary spastic paraplegia
Source: Brain. 2019 Oct 22;142(11):3382–97. doi: 10.1093/brain/awz291 (PMC6821184; doi:10.1093/brain/awz291)
Supplement: awz291_Supplementary_Data [file awz291_supplementary_data.zip › awz291-Suppl_data/Supplementary_Data4.zip!/Supplementary information 3 - fibroblast lipidomics.zip!/Fibroblast lipidomics/Patient1 vs Patient2/Statistical plots and data/Heatmap sorted on p-value.pdf]

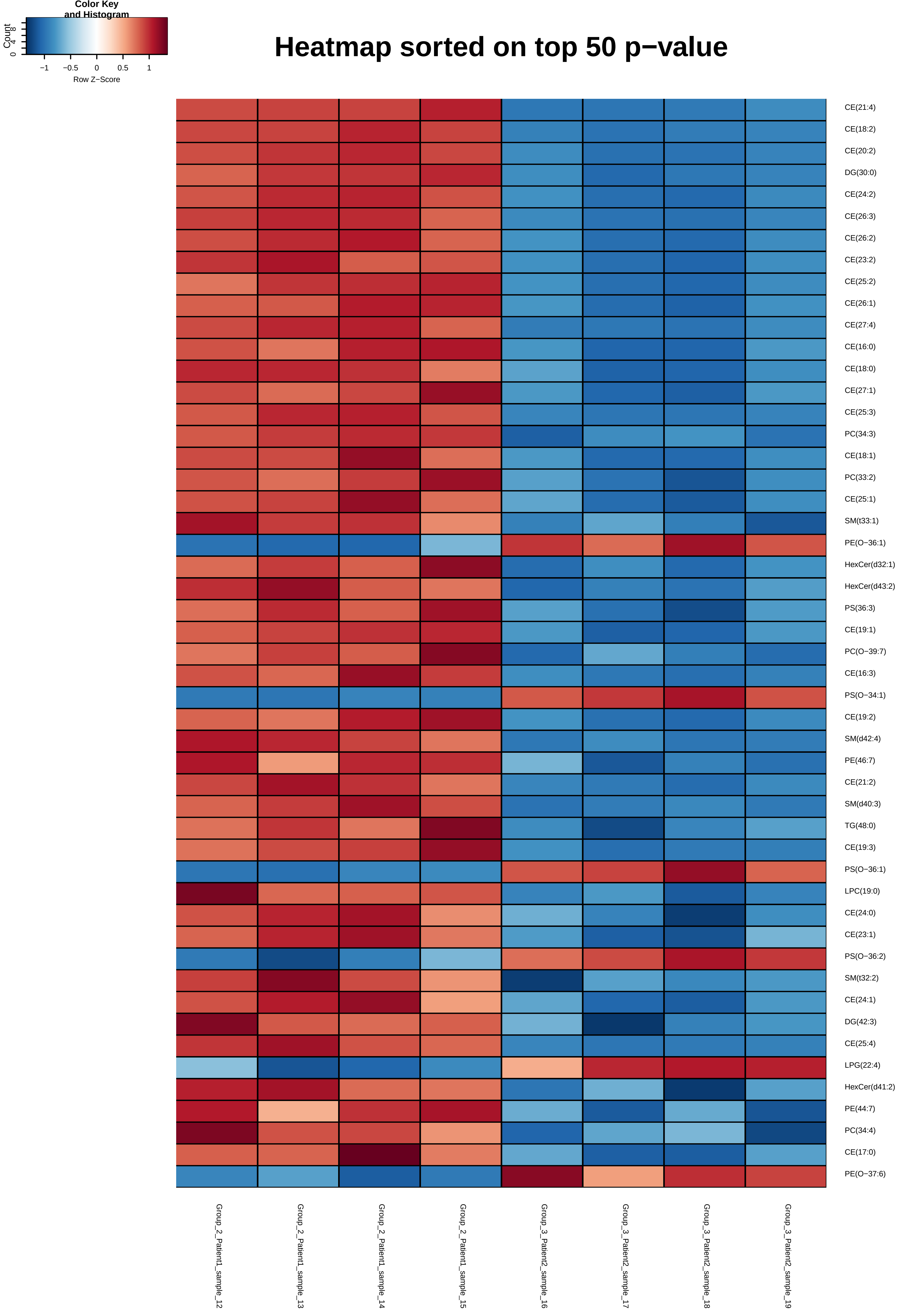
Color Key
and Histogram
Count
0
4
8
−1
−0.5
0
0.5
1
Row Z−Score
Heatmap sorted on top 50 p−value
CE(21:4)
CE(18:2)
CE(20:2)
DG(30:0)
CE(24:2)
CE(26:3)
CE(26:2)
CE(23:2)
CE(25:2)
CE(26:1)
CE(27:4)
CE(16:0)
CE(18:0)
CE(27:1)
CE(25:3)
PC(34:3)
CE(18:1)
PC(33:2)
CE(25:1)
SM(t33:1)
PE(O−36:1)
HexCer(d32:1)
HexCer(d43:2)
PS(36:3)
CE(19:1)
PC(O−39:7)
CE(16:3)
PS(O−34:1)
CE(19:2)
SM(d42:4)
PE(46:7)
CE(21:2)
SM(d40:3)
TG(48:0)
CE(19:3)
PS(O−36:1)
LPC(19:0)
CE(24:0)
CE(23:1)
PS(O−36:2)
SM(t32:2)
CE(24:1)
DG(42:3)
CE(25:4)
LPG(22:4)
HexCer(d41:2)
PE(44:7)
PC(34:4)
CE(17:0)
PE(O−37:6)
Group_2_Patient1_sample_12
Group_2_Patient1_sample_13
Group_2_Patient1_sample_14
Group_2_Patient1_sample_15
Group_3_Patient2_sample_16
Group_3_Patient2_sample_17
Group_3_Patient2_sample_18
Group_3_Patient2_sample_19
